# Is There a Governing Role of Osteocytes in Bone Tissue Regeneration?

**DOI:** 10.1007/s11914-020-00610-6

**Published:** 2020-07-17

**Authors:** Wei Cao, Marco N. Helder, Nathalie Bravenboer, Gang Wu, Jianfeng Jin, Christiaan M. ten Bruggenkate, Jenneke Klein-Nulend, Engelbert A. J. M. Schulten

**Affiliations:** 1 Department of Oral Cell Biology, Academic Centre for Dentistry Amsterdam (ACTA), University of Amsterdam and Vrije Universiteit Amsterdam, Amsterdam Movement Sciences, Amsterdam, The Netherlands; 2 Department of Oral and Maxillofacial Surgery/Oral Pathology, Amsterdam University Medical Centers and Academic Centre for Dentistry Amsterdam (ACTA), Vrije Universiteit Amsterdam, Amsterdam Movement Sciences, De Boelelaan 1117, 1081 HV Amsterdam, The Netherlands; 3 Department of Clinical Chemistry, Amsterdam University Medical Centers, Vrije Universiteit Amsterdam, Amsterdam Movement Sciences, Amsterdam, The Netherlands; 4 Department of Oral Implantology and Prosthetic Dentistry, Academic Centre for Dentistry Amsterdam (ACTA), University of Amsterdam and Vrije Universiteit Amsterdam, Amsterdam Movement Sciences, Amsterdam, The Netherlands; 5 Laboratory for Myology, Faculty of Behavioral and Movement Sciences, Vrije Universiteit Amsterdam, Amsterdam Movement Sciences, Amsterdam, The Netherlands

**Keywords:** Bone regeneration, Bone tissue engineering, Bone remodeling, Mechanical loading, Osteocyte

## Abstract

**Purpose of Review:**

Bone regeneration plays an important role in contemporary clinical treatment. Bone tissue engineering should result in successful bone regeneration to restore congenital or acquired bone defects in the human skeleton. Osteocytes are thought to have a governing role in bone remodeling by regulating osteoclast and osteoblast activity, and thus bone loss and formation. In this review, we address the so far largely unknown role osteocytes may play in bone tissue regeneration.

**Recent Findings:**

Osteocytes release biochemical signaling molecules involved in bone remodeling such as prostaglandins, nitric oxide, Wnts, and insulin-like growth factor-1 (IGF-1). Treatment of mesenchymal stem cells in bone tissue engineering with prostaglandins (e.g., PGE_2_, PGI_2_, PGF_2α_), nitric oxide, IGF-1, or Wnts (e.g., Wnt3a) improves osteogenesis.

**Summary:**

This review provides an overview of the functions of osteocytes in bone tissue, their interaction with other bone cells, and their role in bone remodeling. We postulate that osteocytes may have a pivotal role in bone regeneration as well, and consequently that the bone regeneration process may be improved effectively and rapidly if osteocytes are optimally used and stimulated.

## Introduction

Bone tissue regeneration plays an increasingly important role in contemporary clinical treatment [1]. The reconstruction of bone defects remains a huge challenge for clinicians. Bone defects may result from different causes, such as systemic or local causes. Systemic causes contain congenital abnormalities, general diseases, and effect of medicine, while local causes include inflammation, trauma, or surgical treatments [1]. Autologous bone grafting is still considered the “gold standard” for repair and reconstruction of skeleton [1]. However, autologous bone grafts have the shortcomings, such as limited amount of graft tissue and donor site morbidity. The regeneration of lost bone tissue, thereby recovering bone’s functionality, is challenging. The clinical need for a bone graft with a good quantity (volume) and quality (bone structure) is becoming more urgent. With the continuous development and improvement of tissue engineering technology, bone tissue regeneration will likely become an effective treatment. A better understanding of the mechanisms in bone tissue engineering will eventually result in successful bone regeneration. Therefore, new mechanisms of stimulation of bone regeneration to achieve optimal healing of bone defects may be the key topic of future treatment of bone defects.

In bone tissue, osteocytes encompass approximately 90–95% of the bone cells [2]. In recent years, a big improvement in concept and technology in many fields has helped to interpret the function of osteocytes in bone metabolism and the mechanisms they use to perform their function. Now osteocytes are recognized as the major orchestrator of bone homeostasis, including mechanical sensing and transducing mechanical signals into chemical signals through its lacuna-canalicular system to regulate both bone formation and resorption during bone remodeling [2]. Bone remodeling occurs throughout life [3]. It is a crucial process to maintain a balance of bone homeostasis. Being the master orchestrator of bone, the osteocyte regulates bone remodeling in direct and indirect ways. On the one hand, osteocytes can feel mechanical stimuli and stress changes and regulate matrix remodeling directly [4]. On the other hand, osteocytes orchestrate the activity of osteoclasts and osteoblasts, thereby indirectly regulating bone resorption and bone formation, resulting in a balance of bone homeostasis [4].

Osteocytes play a crucial role in bone (re)modeling. Since mature osteocytes are embedded in mineralized, hard matrix, and therefore difficult to study, the function of the osteocyte in bone tissue engineering is, so far, largely unknown. We postulate that the osteocyte may have a pivotal role in bone regeneration as well and, consequently, that the bone regeneration process may be improved effectively and rapidly if osteocytes are optimally used and stimulated.

## Osteocytes Governing Bone Remodeling

**Fig. 1:**
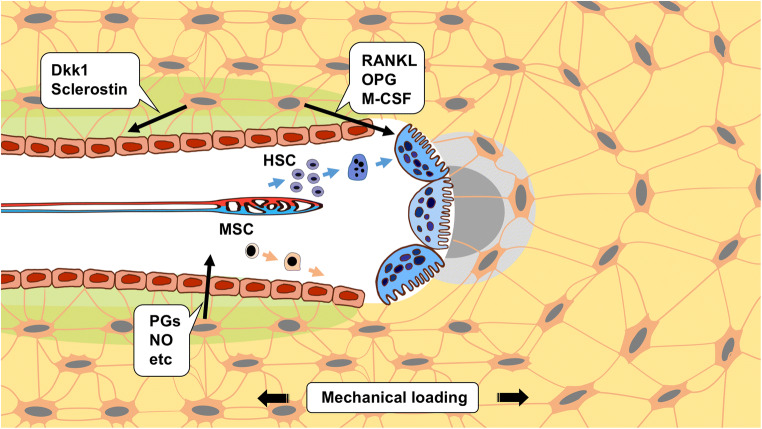
Schematic representation of the role of osteocytes in bone remodeling. (1) Accumulation of fatigue microdamage (gray matrix) interferes with canalicular fluid flow and osteocyte signaling by disrupting canaliculi and damaging osteocyte processes. (2) Following mechanosensation and conversion of the mechanical signal into a chemical signal, osteocytes orchestrate the formation and/or activity of osteoblasts and osteoclasts. PGs, prostaglandins; NO, nitric oxide; Dkk1, Dikkopf-1; RANKL, receptor activator of NF-kappa B ligand; OPG, osteoprotegerin; M-CSF, macrophage-colony stimulating factor

The bone remodeling cycle is a complicated process that starts when fatigue microdamage is sensed by the osteocytes followed by their signaling to the bone surface (Fig. 1). This microdamage eventually disrupts canaliculi resulting in osteocyte apoptosis, and stimulates differentiation of hematopoietic stem cells into osteoclast precursors, and subsequently attachment of multinucleated osteoclasts to the bone surface. Then, bone resorption occurs, accompanied by osteoclast signaling to osteoclast precursor cells within the remodeling space. In the early reversal phase, cells of the osteoblast lineage cover the surface of bone and, with small osteoclasts, finish the resorption phase. During the late reversal phase, osteoblast precursor cells stack on the bone surface until enough cells are present to initiate the bone formation phase [5].

The regulation of bone remodeling is both systemically and locally driven [6]. The major factors involved in the systemic regulation include parathyroid hormone (PTH) [7], growth hormone [8], glucocorticoids [9], thyroid hormones [10], and sex hormones [11, 12]. As far as local regulation of bone remodeling is concerned, the local regulators such as insulin-like growth factors (IGFs) [13], prostaglandins [14], and a large number of cytokines and growth factors secreted by osteocytes are involved as well (Table 1).

**Table 1:** Cytokines and growth factors involved in local regulation of bone remodeling produced by osteocytes

Molecule	Function	Attribute	Ref (s)
CD44	Osteocyte processes formation	Receptor	[15]
MMP-14	Canaliculi formation	Cytokine	[16]
PHEX	Phosphate metabolism, matrix mineralization	Cytokine	[17]
MEPE	Phosphate metabolism, matrix mineralization	Cytokine	[18]
FGF-23	Phosphate metabolism, matrix mineralization	Growth factor	[19]
DMP-1	Phosphate metabolism, matrix mineralization	Cytokine	[20]
Dkk1	Inhibition of bone formation	Cytokine	[21]
Sclerostin	Inhibition of bone formation	Cytokine	[22, 23•]
RANKL	Osteoclast differentiation	Receptor activator	[24]
M-CSF	Osteoclast differentiation	Cytokine	[25]
OPG	Inhibition of osteoclast differentiation	Cytokine	[24, 26]
Cx43	Bone remodeling and cellular interconnections	Cytokine	[27]
IGF-1	Regulation of bone mass	Growth factor	[13, 28]
PGs	Modulation of bone quantity and quality	Signaling lipid	[14, 29]
NO	Modulation of osteoblast and osteoclast activity	Free radical	[30, 31••]

*E11*, membrane-associated protein E11; *CD44*, cell-surface receptor 44; *MMP-14*, matrix metalloproteinase 14; *PHEX*, phosphate-regulating protein with homologies to endopeptidases on the X chromosome; *MEPE*, matrix extracellular phosphoglycoprotein; *FGF-23*, fibroblast growth factor 23; *DMP-1*, dentin matrix protein 1; *Dkk1*, Dikkopf-1; *RANKL*, receptor activator of NF-kappa B ligand; *M-CSF*, macrophage-colony stimulating factor; *OPG*, osteoprotegerin; *Cx43*, connexin 43; *IGF-1*, insulin-like growth factor 1; *PGs*, prostaglandins; *NO*, nitric oxide; *Ref (s)*, reference(s)

### Mechanosensing of Osteocytes

Osteocytes are embedded within a hard matrix in bone for life, only being released by fracture or during remodeling. In this mineralized tissue, the osteocyte is bathed in a bone fluid that travels over the cytoplasmic processes and cell bodies creating shear stresses [32]. The osteocyte is thought to sense stresses to induce signals to osteoclasts and osteoblasts to initiate bone remodeling [5].

Mechanosensation and conversion of the mechanical signal into a chemical signal by osteocytes require a collection of cellular proteins [33–35]. The exact mechanism of the primary mechanosensory apparatus is still unknown. Integrin complexes are likely candidates for osteocyte mechanosensors [33–35]. For example, β1 integrin has been shown to initiate the response of osteocytes to fluid flow [33]. Furthermore, integrins such as α5β1 play a role in the detection of mechanical loading by bone cells at different sites and site-specific mechanotransduction affecting bone homeostasis [34]. In cortical osteocytes, β1 integrins mediate specific aspects of mechanotransduction; i.e., these integrins limit changes in cortical geometry in response to disuse [35]. Although the signal-receptor mechanism in osteocytes is still unclear, more and more studies focus on signaling response of osteocytes to mechanical stimulation. Mechanical signals stimulate osteocytes to produce among others PGE_2_ [14], nitric oxide (NO) [30, 31], and growth factors such as insulin-like growth factor-1 (IGF-1) [36•]. Wnt signaling is crucial in osteocyte mechanotransduction [37]. The deletion of Lrp5 [38] or β-catenin [39, 40] in osteocytes or overexpression of sclerostin in the osteocyte [41••] severely impairs mechanotransduction in bone. Taken together, the exact mechanism of mechanosensation by osteocytes is still unknown, but new biological methods and technological advances are fostering progress in it.

### Osteocytes Manipulating their Microenvironment

The osteocyte has the function to remove and renew the matrix around its lacuno-canalicular system. This process of the osteocyte affects the calcium levels in plasma, especially during special physiological periods, such as lactation or hibernation [4]. In skeleton, the matrix is removed by osteoclasts and renewed by osteoblasts. Osteocytes can remodel perilacunar and pericanalicular matrix like osteoblasts expressing the genes necessary for bone formation, as well as expressing specific genes for matrix resorption like osteoclasts [42]. The average number of osteocytes in the adult human bone is approximately 42 billion, and the average number of dendritic projections of these cells is about 3.7 trillion with a total surface area of 215 m^2^ in the lacuno-canalicular system [43]. Therefore, osteocytes may regulate bone resorption by osteoclasts via calcium release, thereby maintaining bone balance.

### Osteocytes Governing Osteoclasts

In order to orchestrate mechanical adaptation of bone structure, osteocytes specifically coordinate the activity of osteoclasts through a variety of mechanisms. Extensive research on the RANKL/osteoprotegerin (OPG) mechanism has been performed to determine the role for osteocyte control of osteoclast biology [44••]. Osteocytes are a major source of RANKL for osteoclastogenesis. It has been shown that selective deletion of the RANKL gene in osteocytes of engineered mice accounts for the deficient osteoclastogenesis phenotype observed in global mutants [26, 45]. Osteocytes are also important for the production of OPG, which inhibits osteoclast formation by acting as a soluble decoy receptor for RANK [26]. Thus, osteocytes control the formation of osteoclasts by stimulation of RANKL expression and/or availability, and by inhibition of OPG expression and/or availability. It is also possible that the proportions can be reversed to diminish bone resorption.

Osteocytes control osteoclastogenesis. They are capable of chemically attracting remodeling units into bone that has to be renewed [21, 22, 24, 25, 46–50]. New remodeling BMUs (teams of osteoclasts and osteoblasts that resorb and deposit bone in a coordinated manner while moving through the tissue space) have been shown to be highly associated with fatigue-induced microcracks; i.e., the association is 4 to 6 times more likely than by chance alone in canine bone [46]. Severe cortical bone microdamage results in osteocyte apoptosis, which initiates bone remodeling [47]. It has been demonstrated that apoptotic osteocytes in fatigue damaged regions signal healthy osteocytes at about 200 μm distance to produce RANKL, which in the end is of use to attract remodeling units. The signals from apoptotic osteocytes to cause the release of RANKL by nearby cells entail ATP signaling via Panx1 and P_2_X_7_ activation [24]. Other factors that are derived from osteocytes and contribute to osteoclast differentiation and function include macrophage-colony stimulating factor (M-CSF) [25], interleukin-6 (IL-6) [48], tumor necrosis factor-α (TNF-α) [22], perhaps through osteocyte-derived apoptotic bodies [49], and high mobility group box 1 (HMGB1) [21]. During the last years, the understanding of osteocyte control of osteoclastogenesis has substantially increased, while currently, research is performed on several RANKL/OPG independent mechanisms [50].

### Osteocytes Governing Osteoblasts

Osteocytes regulate osteoblasts in both indirect and direct ways [4]. Most indirect effects of osteocytes on osteoblasts may be based on the coupling phenomenon in the process pf bone remodeling. Osteocytes regulate the activity of osteoclasts (bone resorption) in direct way, whereas osteocytes regulate the activity of osteoblasts (bone formation) in indirect way [4]. However, the osteocyte also regulates the osteoblast directly which occur via the production of both inhibitory and stimulatory factors [29, 51–58].

Osteocyte-produced inhibitory factors include the Lrp5/6 antagonists sclerostin and Dikkopf-1 (Dkk1) [51]. Neuropeptide Y is another crucial osteocyte-derived factor in osteoblast activity. Osteocytes demonstrate high expression of neuropeptide Y that causes inhibition of osteoblast activity [52]. Osteocytes are the major source for sclerostin, although sclerostin is expressed in several tissues other than bone [53]. Osteocytes also demonstrate high expression of Dkk1 [51]. Sclerostin and Dkk1 are strong antagonists of Wnt-mediated activity in osteoblasts [51, 53]. Some anabolic stimuli such as mechanical loading [54], PTH [55], and PGE_2_ [29] reduce sclerostin expression in osteocytes, which in the end expedites osteoblast-mediated anabolism through Wnt. Regarding osteocyte-produced stimulatory factors, osteocytes are a rich source of PGE_2_ (signaling lipid) [14], IGF-1 (growth factor) [36•], Wnts (glycoproteins) [56•, 57], NO (free radical) [30], and ATP (nucleotide) [58] that have potent effects on osteoblastogenesis and matrix formation.

In the context of bone diseases, much research so far on the communication between osteocytes and osteoblasts has focused on the control of inhibitors of osteoblasts that are derived from osteocytes [51, 53]. However, osteocyte-derived molecular activators of osteoblast function are likely equally important in the remodeling process and may be key players in both bone remodeling and bone regeneration.

## Osteocytes Governing Bone Regeneration

The current approaches using bone graft substitutes for bone tissue regeneration are (1) synthetic scaffolds alone, (2) scaffolds combined with active molecules, and (3) cell-based combination products with stem cells from various sources [59]. The most appropriate approach for bone regeneration to reconstruct a bone defect depends partly on the size of the defect. If a bone defect is small (defect size: < 2 cm), only scaffolds can be used in clinical treatment. If a bone defect is intermediate (defect size: 2 to 4 cm), bioactive molecules, such as BMP-2 or TGF-β, can be combined with scaffolds to improve bone regeneration. If a bone defect is large (defect size: > 4 cm), there is a need to mix cell-based combination products with stem cells into the scaffold to enhance biological functionality in bone defect repair [59].

Cell-based bone tissue engineering techniques utilize both stem cells and biomedical materials and have emerged as a promising approach for large volume bone repair. Here, a sufficient cell population for the therapy needs to be insured, which might require cell expansion. Thereafter, cells can be stimulated to induce osteogenic differentiation followed by seeding into a biomaterial containing stimulatory molecules such as growth factors. The fate of the implanted cells likely depends on the cell type, differentiation stage, and stimulatory factors used, in combination with the biomaterial [60].

The unresolved clinical challenges of bone tissue regeneration are related to the limited healing capacity of bone tissue, since current bone tissue regeneration cannot cover all types of bone defects, especially when the bone defect is large and complex. Bone tissue regeneration can be improved if we better understand the exact mechanism that bioactive molecules have on cells involved in bone tissue regeneration [61]. Moreover, detailed knowledge of the microenvironment at the defect site and how this affects and interacts with cells in the engineered bone is needed. Importantly, the properties of the reconstructive scaffold should closely match and/or actively modify the patient microenvironment towards the optimal regeneration niche. Development of combined bone repair strategies targeting growth factor receptors and cellular adhesion receptors to stimulate cooperative signaling and optimize bone repair is needed. The current mode of delivery of bioactive molecules in vivo has to be further explored, whereby the exact control and confinement of the bioactive molecules is warranted to avoid adverse side effects. The dosages of the bioactive molecules used to stimulate bone regeneration require optimization in order to prevent overdosing and also side effects. Moreover, the mechanical properties of the current engineered scaffolds need to be improved for better integration in the bony environment, which improves the success rate of large bone defect repair via modulation of the scaffold, and coupling of different cell types (e.g., osteocytes, endothelial cells) with stem cells.

Osteocytes play a role in different types of bone regeneration, including matrix regeneration (osteocytic osteolysis) and fracture healing. First, osteocytic osteolysis is referring to the pathologic removal of the perilacunar matrix as occurs with diseases such as hyperparathyroidism, hypophosphatemic rickets, and osteoporosis [4]. Osteocytes likely use a similar molecular mechanism as osteoclasts to remove mineral, since calcium release from mineralized bone requires a low pH and specialized enzymes. Osteocytes can reverse the osteolytic process by replacing the removed matrix. Thus, osteocytes can acidify their lacunar-canalicular space to demineralize the matrix by producing protons via the action of carbonic anhydrase-2, and releasing protons via proton pumping by vacuolar ATPases. The organic components are removed from the perilacunar matrix via the actions of MMP-13, tartrate resistance acid phosphatase, and cathepsin K. Osteocytic osteolysis can be induced by, e.g., activation of PTHR1 by PTH, PTHrP, and TGF-β signaling, and by increased sclerostin production as a result of disuse [3].

Second, osteocytes play a role during fracture healing, from the early to the late phase. At the early phase, osteocytes located close to the fracture site become apoptotic. Proinflammatory factors, e.g., interleukin 6 (IL-6) and cyclooxygenase-2 (COX-2), are upregulated, which stimulates the coordinated bone healing response at the inflammatory stage. Growth factors, e.g., bone morphogenetic protein-2 (BMP-2), are expressed to promote revascularization and neoangiogenesis of callus tissue. Osteogenesis is stimulated via increased expression of osteocyte-specific markers E11 and dentin matrix protein 1 (DMP-1) and decreased expression of sclerostin. At the intermediate phase of fracture healing, osteocytes still express growth factors, e.g., BMP-2 and cysteine-rich angiogenic inducer 61 (CYR61), leading to soft callus formation and chondrogenesis. BMP-2 expression decreases with progression of healing. E11 and Cx43 are upregulated for the maintenance of the lacuno-canalicular network. E11/gp38 is expressed as osteoblasts differentiate into osteocytes, and regulates osteocyte dendrite formation and elongation. Cx43 expression stimulates intercellular communication between osteocytes, modulates osteoblast signaling, and aids osteocyte survival. DMP-1 expression indicates osteocyte maturation and mineralization. Sclerostin expression restores to its normal level to suppress osteoblastic action. At the late phase, healing continues with remodeling and mineralization of the bony callus. After re-establishment of the lacuna-canalicular network, expression of DMP-1, E11, and Cx43, but not sclerostin, decreases, indicating osteocyte maturation. Mineralized bone matrix-embedded osteocytes express matrix extracellular phosphoglycoprotein (MEPE) which persisted expression might indicate a role in the rapid callus mineralization at the late phase of fracture healing [62].

**Fig. 2:**
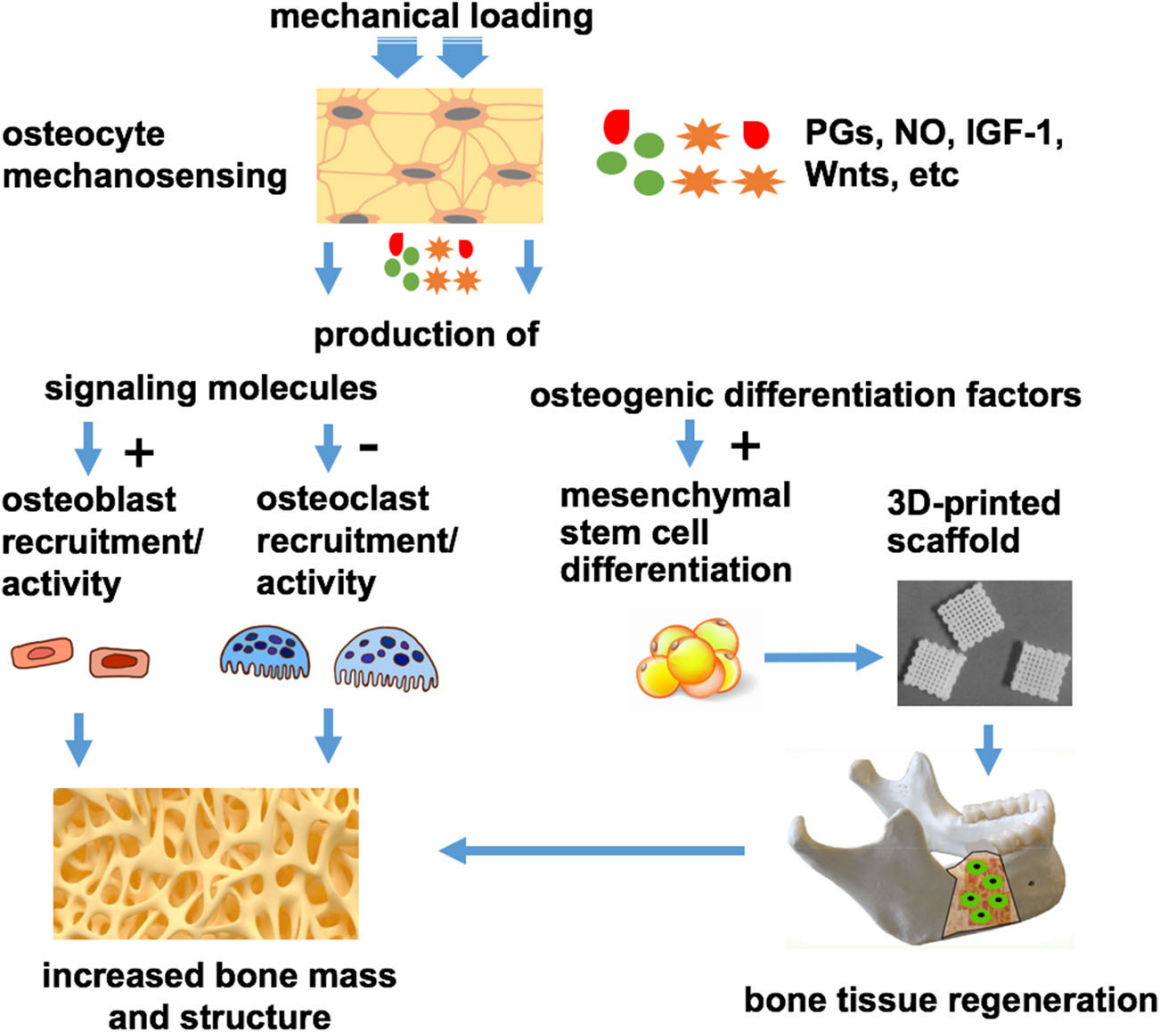
Overview of the role of osteocytes in bone tissue regeneration. Several signaling molecules, such as prostaglandins, NO, Wnts, and IGF-1, are secreted by osteocytes after mechanical stimulation. These signaling molecules not only regulate bone homeostasis but also affect osteogenic differentiation of stem cells. PGs, prostaglandins; NO, nitric oxide; IGF-1, insulin growth factor-1; 3D, 3-dimensional

Osteocytes orchestrate bone formation and bone resorption, following mechanosensation and mechanotransduction, i.e., the conversion of a mechanical stimulus into a chemical signal. This complex process is driven by several biomolecules, such as prostaglandins, NO, Wnts, and IGF-1 [63]. Interestingly, these biomolecules released by osteocytes are also found to be actively involved in bone regeneration processes. Bone tissue regeneration aims to treat bone defects and is the goal of bone tissue engineering, in which engineered scaffolds, bioactive molecules, and stem cells are involved [64]. These stem cells play an important role in bone tissue regeneration. Therefore, osteocytes are likely governing bone regeneration via the production of effective factors regulating osteogenic differentiation of stem cells (Fig. 2).

Prostaglandins, such as PGE_2_, PGI_2_, and PGF_2α_, are produced by osteocytes as well as other bone cells in response to, e.g., mechanical stimulation, and play an important role during bone remodeling [65]. The key enzyme involved in prostaglandin production is cyclooxygenase (COX) [65]. Both in vitro and in vivo, mechanical loading causes a rapid increase in COX-2 mRNA and protein in osteocytes [66]. COX-2 mediates the anabolic response of bone tissue to mechanical loading, which shows that prostaglandin production as a result of mechanical loading is crucial for adaptive bone remodeling [67]. COX-2 might therefore also be of importance for bone regeneration. PGE_2_ is the most abundant prostaglandin in bone. At low concentrations, PGE_2_ stimulates osteoblast differentiation and increases bone formation, but at high concentrations, it stimulates bone resorption and inhibits collagen synthesis [68]. PGF_2a_ stimulates alkaline phosphatase (ALP) gene expression and activity of the bone extracellular matrix proteins osteopontin (OPN) and α1(Ι) procollagen (COL1A1) in human adipose tissue–derived mesenchymal stem cells, which suggests that these cells undergo osteogenic differentiation after treatment with PGF_2a_ [68]. This is also a crucial process in bone regeneration.

NO plays a critical role in bone remodeling as a chemical signal [69]. Activated osteocytes produce signaling molecules like NO, which modulate the activity of osteoblasts and osteoclasts, thereby orchestrating bone adaptation to mechanical loading [69]. Endogenous NO deficiency causes some pathological conditions such as osteoporosis, pointing to a role of NO in bone formation [70]. In contrast, NO counteracts bone loss [71]. Therefore, exogenous NO supplementation seems a probable strategy for bone regeneration to treat osteoporosis. Osteogenic differentiation of stem cells is regulated by NO [70]. Exogenous NO supply to stem cells enhances osteogenic differentiation [71]. In addition, NO supplementation in a controlled manner in cell-based therapeutics may be an excellent strategy to improve the function of stem cells [72]. These reports indicate that NO can be used for regulating osteoblastic differentiation and bone formation.

Wnt proteins are secreted growth factors with pivotal roles in many cellular activities, including proliferation, migration, and differentiation. They also play an important regulatory role in the process of perilacunar/canalicular remodeling, as mediated by osteocytes [37]. Moreover, they are important in regulating the balance of osteogenesis mediated by osteoblasts and bone resorption mediated by osteoclasts at the bone surface, at least in part through the canonical Wnt/β-catenin signaling pathway and the OPG/RANKL signaling pathway [37]. Wnts bind to receptors of the Frizzled family [37]. This complex connects with membrane-associated proteins, such as low-density lipoprotein receptor–related proteins 5 and 6 (LRPs) [37], hereby activating the canonical signaling pathway [56•]. In MLO-Y4 osteocytes, pulsating fluid flow upregulates gene expression of Wnt3a, c-jun, connexin-43, and CD44, suggesting an upregulation of Wnt signaling [57]. Wnt signals both in vivo and in vitro carry the potential for therapeutic approaches such as tissue engineering for regenerative medicine [73].

IGF-1 is another important factor for the cellular response to mechanical loading in osteocytes and human adipose stem cells [74, 75]. The IGF system comprises the receptors (type I and type II), the ligands (IGF-1 and IGF-2), IGF binding proteins (IGFBPs), and IGFBP proteases [13]. These proteins promote cell survival, proliferation, and differentiation, and thus mediate the stimulation of somatic growth [76]. Osteocytes highly express IGF-1 [77]. IGF-1 and IGF-1R (IGF-1 receptor) are key players in the development of the human embryonic skeleton and in obtaining peak bone mass during postnatal growth [13]. IGF-1 signaling is crucial in the bone response to mechanical stimuli [78]. Mechanically stimulated rat tibia in vivo and mechanically stimulated osteocytes in vitro demonstrate enhanced IGF-1 expression after one bout of increased mechanical loading [74]. IGF-1 is a crucial factor in that it modulates PTH/PTHrP receptor signaling in osteocyte-controlled periosteal bone formation and intracortical remodeling [79, 80]. IGF-1 induces osteogenic differentiation of human mesenchymal stem cells in vitro, suggesting that it might be an alternative for bone morphogenetic protein-7 [28]. IGF-1 supplementation significantly enhances de novo vasculogenesis in vitro and in vivo [81]. Thus, the methodology of IGF-1 supplementation is highly promising for engineering de novo vasculature in tissue regeneration [81]. Three-dimensional (3D) culture of mesenchymal stem cells with 3D titanium scaffolds enhances osteogenic differentiation and new bone formation through the IGF-1R/AKT/mTORC1 pathway [82]. This method of osteointegration may have clinical application in the preparation of bone grafts before implantation to improve the repair of mandibular bone defects [82].

In summary, osteocytes secrete many molecules affecting bone metabolism (Table 1). Not only prostaglandins, NO, Wnts, and IGF-1 but also other molecules such as E11, CD44, and MMP-14 (related to osteocyte processes formation and canaliculi morphology) [15, 16, 83], as well as PHEX, MEPE, FGF-23, and DMP-1 (related to phosphate metabolism and matrix mineralization) [17–20], are produced. Several factors such as prostaglandins, NO, Wnts, and IGF-1 secreted by osteocytes regulate stem cell differentiation and osteogenesis. Thus, it may be expected that the production of these factors by osteocytes might play an important and likely even governing role in bone regeneration as well. Fine-tuning the bone formation-promoting secretome of the osteocyte towards an optimal bone-regenerating repertoire of growth factors may be an important new strategy in bone regeneration efforts.

To further interpret the role of osteocytes in bone regeneration, different cell compositions could be investigated in an in vitro human bone regeneration model. For example, osteocytes could be co-cultured with stem cells and mixed in calcium phosphate scaffolds to improve bone regeneration. The biochemical signaling molecules, which are secreted by osteocytes, could also be investigated regarding their capacity to repair bone defects in an in vitro human bone regeneration model. Such experiments help to elucidate blood vessel and bone cell interactions during bone development and repair, and the results can be extrapolated to clinical application for bone defect reconstruction. This in vitro human bone regeneration model allows to test different scaffold materials and peptides for their vasculogenic and/or osteogenic properties. Moreover, this model allows in vitro pre-screening of patients with insufficient bone volume for dental implant placement to predict the bone regeneration capacity in vivo resulting from the bone augmentation procedure.

## Conclusions

Osteocytes have multiple functions in bone tissue. They can sense fluid shear stress as a result of mechanical loading, translating the mechanical stress into the production of biochemical signaling molecules. These effective biomolecules further regulate bone resorption and bone formation. This internal regulation may not only apply to bone remodeling but is also potentially useful for bone regeneration. Thus, it may well be that osteocytes not only regulate bone turnover but are also able to enhance osteogenesis of stem cells, suggesting a novel yet unrecognized role of osteocytes in governing bone tissue regeneration. The role of osteocytes in bone tissue regeneration should be further explored in future research.
